# Electrophysiological Correlates of Amnestic Mild Cognitive Impairment in a Simon Task

**DOI:** 10.1371/journal.pone.0081506

**Published:** 2013-12-05

**Authors:** Jesús Cespón, Santiago Galdo-Álvarez, Fernando Díaz

**Affiliations:** Department of Clinical Psychology and Biological Psychology, University of Santiago de Compostela, Santiago de Compostela, Galiza, Spain; University of Granada, Spain

## Abstract

Amnestic mild cognitive impairment (aMCI) represents a prodromal stage of Alzheimer`s disease (AD), especially when additional cognitive domains are affected (Petersen et al., 2009). Thus, single-domain amnestic MCI (sdaMCI) and multiple-domain-amnestic MCI (mdaMCI) biomarkers are important for enabling early interventions to help slow down progression of the disease. Recording event-related potentials (ERPs) is a non-invasive and inexpensive measure of brain activity associated with cognitive processes, and it is of interest from a clinical point of view. The ERP technique may also be useful for obtaining early sdaMCI and mdaMCI biomarkers because ERPs are sensitive to impairment in processes that are not manifested at behavioral or clinical levels. In the present study, EEG activity was recorded in 25 healthy participants and 30 amnestic MCI patients (17 sdaMCI and 13 mdaMCI) while they performed a Simon task. The ERPs associated with visuospatial (N2 posterior-contralateral – N2pc -) and motor (lateralized readiness potential – LRP –) processes were examined. The N2pc amplitude was smaller in participants with mdaMCI than in healthy participants, which indicated a decline in the correlates of allocation of attentional resources to the target stimulus. In addition, N2pc amplitude proved to be a moderately good biomarker of mdaMCI subtype (0.77 sensitivity, 0.76 specificity). However, the LRP amplitude was smaller in the two MCI groups (sdaMCI and mdaMCI) than in healthy participants, revealing a reduction in the motor resources available to execute the response in sdaMCI and mdaMCI patients. Furthermore, the LRP amplitude proved to be a valid biomarker (0.80 sensitivity, 0.92 specificity) of both amnestic MCI subtypes.

## Introduction

The pathophysiological processes involved in Alzheimer’s disease (AD) are thought to take place before development of dementia [1]. However, clinical diagnosis of AD is usually made once a patient has developed impairment in multiple cognitive domains that are sufficient to interfere with social routine and/or occupational function.

The concept of Mild Cognitive Impairment (MCI) was developed in order to identify people showing symptoms that are suggestive of AD but that are not sufficiently severe to interfere in lifestyle [2], [3]. Neuropathological [4] and electroencephalographic (EEG) data [5] support the hypothesis that MCI may represent a preclinical stage of AD [6]. Indeed, it has been shown that a high percentage of MCI patients develop dementia within a few years [7]. Thus, MCI markers would constitute good indicators for early treatment [6], which should slow down progression of the disease [8].

Petersen et al. (1999) [3] established a set of criteria to diagnose people suffering from MCI (subjective memory complaint, memory impairment, intact general cognitive functioning, preserved activities of daily living, and not demented). However, it was found that MCI sufferers vary considerably in clinical symptoms and prognosis. In light of this evidence, the concept of MCI was refined by distinguishing MCI subtypes according to presence/absence of episodic memory impairment (amnestic/non-amnestic) and number of affected cognitive domains (single-domain/multiple-domain) [7], [9]. As a result, four MCI subtypes were distinguished: single-domain amnestic MCI (sdaMCI, characterised by only memory impairment), multiple-domain amnestic MCI (mdaMCI, characterised by memory impairment and impairment in other additional cognitive domains), single-domain non-amnesic MCI (sdn-aMCI, characterised by preserved memory but an overt decline in another cognitive domain), and multiple-domain non-amnestic MCI (mdn-aMCI, characterised by preserved memory but with evidence of decline in several cognitive domains).

Studies have shown that the amnestic MCI subtypes are more likely to progress to AD than the non-amnestic MCI subtypes [10], and the prognosis is even worse if amnestic decline is accompanied by impairment in other cognitive functions [11]. In light of this evidence, the present study focused on the search for biomarkers in the two amnestic MCI subtypes (i.e. sdaMCI and mdaMCI), which are more likely to progress to AD.

Several studies have highlighted the existence of valid biomarkers of the MCI state [6], [12]; however, such biomarkers are expensive (fMRI) and invasive (e.g. positron emission tomography (PET) and cerebrospinal fluid measures). On the contrary, recording EEG and event-related potentials (ERPs) is a suitable method for obtaining MCI biomarkers, since it is a widely diffused, non-invasive and relatively inexpensive procedure [13]. In addition, temporal resolution of ERPs is also especially useful for addressing the speed of cognitive processes in order to establish differences in brain electrical measures between MCI and normal ageing. Another pertinent characteristic of the ERP technique is that it enables detection of abnormalities that are not detectable at clinical or behavioral levels [14]. This is of particular interest in the search for very early biomarkers of AD.

The study of ERP correlates of some cognitive processes might be of particular interest for distinguishing the two subtypes of MCI patients from healthy participants on the basis of brain electrical activity. Evidence has been obtained regarding the early impairment of spatial and attentional processes in the progression from normal ageing to AD [15]. In addition, the progressive slowing of reaction time (RT) with increasing age has been attributed to slowing of the motor generating system [16]. Considering that RT is usually longer at very early stages of AD [17], ERP correlates of motor processes may be sensitive to the amnestic MCI states.

The posterior contralateral negativity (N2pc) is an ERP component that has been related to visuospatial processing of a target stimulus [18]. N2pc appears contralaterally to the visual hemifield in which the target is located, 200–300 ms after the onset of a bilateral stimuli array [18]–[21]. The N2pc latency has proved to be a reliable measure of the attentional shift to possible targets [21], [22], whereas the N2pc amplitude reflects the amount of attention that is allocated to a stimulus [23].

Previous studies have shown an age-related slowing in the allocation of attentional resources to the target stimulus (revealed by a longer N2pc latency) in visual search tasks [24], [25] as well as Simon tasks [26]. The N2pc amplitude was also smaller in elderly than in younger participants during visual search tasks [24], [25] although no differences were found in another study [27]. As concluded in the review by Iachini et al (2009) [15], attentional and spatial deficits are expected to appear at very early stages of dementia, so that evaluation of visuospatial processes is considered as a promising approach in the search for predictive markers of AD. However, as far as we know, no previous studies have evaluated the N2pc activity in any MCI subtype and/or AD patients.

The age-related slowing in motor processes was mainly located at the response execution stage, as revealed by studies examining the response-locked lateralized readiness potential (LRP-r) [28]–[32]. However, so far, no studies have focused on LRP component in samples of MCI and/or AD patients. Considering a possible slowing in RT in amnestic MCI patients in comparison to healthy elderly subjects, along with impairment in primary motor regions [33], changes associated with amnestic MCI subtypes in response execution stage should be investigated. Moreover, larger LRP amplitudes were observed in healthy older participants than in young participants [28], [29], [31], which suggested a higher activation threshold of the motor cortex to execute the response in elderly participants. In this context, larger LRP amplitudes were associated with less successful inhibitory control [30], [34]. Given that amnestic MCI patients showed decreased inhibitory control in several studies [35], [36], differences in LRP amplitude between the two groups of amnestic MCI patients and healthy participants may be expected.

In the present study, EEG activity was recorded while participants performed a Simon task. In Simon tasks, participants respond to a non spatial feature of a lateralized stimulus while they have to ignore the stimulus position (for reviews on the Simon task, see Leuthold (2011) [37] and Proctor et al., 2005 [38]). This paradigm enables the study of visuospatial processing of the lateralized stimulus as well as executive (response-related) processes. The aim of the present study was to explore differences in brain electrical activity between healthy participants and the two subtypes of amnestic MCI patients (i.e. sdaMCI and mdaMCI), in order to obtain possible ERP biomarkers. Therefore, the present study focused on N2pc and LRP-r components.

Deficits in spatial abilities are expected to appear at very early stages in the progression from normal ageing to AD. Several studies have suggested that impaired visuospatial abilities may take place even before typical deficits in cognitive memory [39], [40]. Therefore, mdaMCI patients may differ from healthy participants in N2pc parameters; in addition, those participants who only display memory deficits (i.e., the sdaMCI group) may also show differences in N2pc parameters, which would reveal an incipient decline in visuospatial processes in the absence of clinical/behavioural symptoms. Specifically, delays in N2pc latency and/or reductions in N2pc amplitude (related to delayed and reduced allocation of attentional resources to the processing of the target stimulus, respectively) may be expected in the amnestic MCI groups relative to healthy participants.

Regarding motor processes, a lengthening of the response execution stage in the two amnestic MCI groups relative to healthy participants was expected, which would be indicated by earlier LRP-r onset in sdaMCI and mdaMCI groups than in healthy elderly. Differences between healthy participants and participants belonging to both amnestic MCI groups may also be found in LRP amplitudes as a consequence of differences in motor areas for implementing motor resources to execute the response.

## Methods

### Participants

Fifty-five participants (25 women, 30 men) between 51 and 85 years of age (mean age 66.8 years) were recruited from the general population. The participants were divided into 2 groups according to Diagnosis: Control Group (CG (25 participants: 11 women, 14 men), Age Mean: 65.0 (SD: 8.1)), single-domain amnestic MCI (sdaMCI (17, participants: 7 women, 10 men), mean age: 67.0 (9.1)) and multi-domain amnestic MCI group (mdaMCI (13 participants; 7 women, 6 men), mean age: 71.0 (SD: 9.2)). The participants volunteered to take part in the study, which received prior approval by the local ethical review board. All the participants were right-handed (evaluated by the Edinburgh Handedness Inventory [41]). All participants had normal or corrected to normal vision, and none had any history of neurological or psychiatric disorders. The study was approved by the USC ethics committee and by the Galicia Clinical Research ethics committee. The participants received an informative protocol informing them about the aims of the research. The procedure and the type of tasks to be carried out in the neuropsychological and EEG sessions, as well as the purposes of the study, were explained to the participants. When the participant was accompanied by a relative, both were present when the tasks and the aims of the research were explained. All participants gave written informed consent prior to their inclusion in the study. All participants were able to sign the written informed consent because participants with signs and/or symptoms of dementia were excluded from the present research. All potential participants who declined to participate were not disadvantaged in any other way by not participating in the study.

All the MCI patients were amnestic MCI patients, since these patients are more likely to develop AD dementia [6]. Amnestic MCI patients were divided into two different groups: the single-domain amnestic MCI (sdaMCI) subtype and the multiple-domain amnestic MCI (mdaMCI) subtype, according to established criteria for distinguishing MCI subtypes [7], [9].

The following tests were used to diagnose the two subtypes of amnestic MCI (i.e., sdaMCI and mdaMCI): an adapted version [42] of the Mini-mental state Examination (MMSE) [43]; an adapted version [44] of the California Verbal Learning Test [45]; the Cambridge examination for mental disorders in elderly (CAMDEX-r) [46]; a questionnaire on subjective memory complaints [47]; the instrumental activities of daily living scale (IADL) [48]; and the Geriatric depression scale (GDS) [49]. Participants also completed a questionnaire with socio-demographic and clinical data. Finally, there were no differences regarding years old and years of schooling based on the diagnosis.

As already mentioned, 17 participants fulfilled criteria for single-domain amnestic MCI (sdaMCI) (only memory functions were declined) and 13 participants fulfilled criteria for multiple-domain amnestic MCI (mdaMCI). All sdaMCI and mdaMCI participants fulfilled the following criteria: (1) memory complaints corroborated by an informant; (2) performance of less than 1.5 standard deviations (SDs) below age norms for the TAVEC; (3) no significant impact on activities of daily living; and (4) without dementia. In addition, regarding general cognitive functioning, the mdaMCI participants scored less than 1.5 SDs below controls with respect to standards of age and years of schooling in the adapted version of the MMSE, and on at least two cognitive subscales of the Spanish version of the CAMCOG-R (a subscale of the CAMDEX-r), which include subscales for specific domains such as attention-calculation, praxis, and executive functioning and is sensitive to MCI detection [50]. All control participants scored higher than the cut-off on memory, general cognitive functioning, and specific cognitive domain tests (demographic and neuropsychological measures of the participants are summarized in Table 1). For an extensive description of the samples, the inclusion/exclusion criteria, the tests used, and the diagnosis and classification criteria, see Juncos-Rabadán et al. (2013) [51].

**Table 1:** Mean values and standard deviations (SD, in parentheses) of the main demographic and neuropsychological measures.

	CG	sdaMCI	mdaMCI
Age	65.2 (8.2)	67.0 (9.1)	71.0 (9.2)
Schooling	10.8 (5.6)	8.9 (4.3)	9.2 (4.8)
WAIS_language	49.7 (16.3)	45.8(13.4)	38.5 (14.4)
CAMCOG_MMSE	28.4 (1.3)	27.2 (1.9)	23.5 (1.7)*
CAMCOG_orientation	9.6 (0.5)	9.4 (0.7)	8.4 (1.2)*
CAMCOG_language	26.3 (2.1)	25.1 (2.3)	23.0 (2.3)*
CAMCOG_calculation	7.7 (1.3)	7.2 (1.9)	5.1 (2.5)*
CAMCOG_praxis	11.4 (1.0)	10.6 (2.5)	9.2 (2.3)*
CAMCOG_perception	6.8 (1.6)	6.2 (1.5)	6.2 (1.6)
CAMCOG_executive	19.0 (5.1)	14.5 (3.8)*	12.9 (3.7)*
CVLT (short delay free recall)	10.0 (2.8)	3.6 (2.2)*	3.4 (2.1)*
CVLT (short delay cue recall)	10.6 (2.6)	4.9 (2.4)*	5.7 (2.4)*
CVLT (long delay free recall)	10.0 (3.3)	4.7 (3.0)*	3.4 (3.3)*
CVLT (long delay cue recall)	11.2 (2.8)	6.4 (2.7)*	5.6 (2.6)*

t-tests were carried out to compare scores at group level on each cognitive scale. Asterisks indicate significant differences (p<0.05) between CG and sdaMCI and between CG and mdaMCI groups. Although group differences between CG and sdaMCI groups were significant in CAMCOG-executive scale, performance on each individual participant was not lower than 1.5 SD for norms of age and years of schooling.

### Task

**Figure 1:**
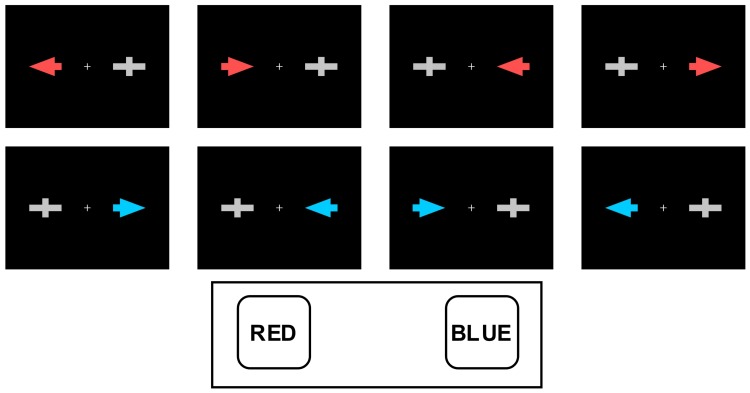
The Simon task with stimuli presented and response buttons. Participants were instructed to respond by pressing the left button with the left hand when a red arrow appeared, and the right button with the right hand when a blue arrow appeared, so that the conditions presented (from left to right) were, respectively, as follows: compatible direction and compatible position (CDCP); incompatible direction and compatible position (IDCP); compatible direction and incompatible position (CDIP), and incompatible direction and incompatible position (IDIP). The response buttons were counterbalanced between participants.

A series of red or blue arrows pointing either left or right was displayed on a screen against a black background. The screen was placed 100 cm in front of the participants. The arrow stimuli subtended 2.87° long and 1.72° wide of the visual field. The visual angle between the central cross on the screen and the internal edge of the arrow was 2.29°, and the visual angle between the cross and the external edge of the arrow was 5.16°, so the entire stimulus was presented in the parafoveal region [52]. A grey geometric figure of similar morphology and eccentric position (two orthogonally superimposed bars, the vertical thicker than the horizontal, see Figure 1) was presented in the opposite hemifield to the target with the aim of preventing exogenous lateralization in the electroencephalogram (EEG). The arrows (and the contralateral geometric figure) were presented for 125 ms, with 2000 ms inter-trial intervals. The participants were instructed to direct their gaze towards the central cross throughout the task; this, along with the short interval during which the stimuli were presented, minimized the likelihood of ocular movements towards the area where the arrow appeared [53].

### Procedure

Participants carried out the task while seated in a comfortable chair in a dimly lit, sound-attenuated, electrically shielded chamber. They were instructed to respond to the colour of the arrow by pressing one of two horizontally positioned buttons (blue or red), but to ignore the position and the direction indicated by the arrow (Figure 1). The arrow was presented on either side of the central cross (where the participants were asked to direct their gaze throughout the task) and pointed either to the left or to the right. The two irrelevant dimensions (position and direction indicated by the arrow) gave rise to four experimental conditions depending on whether they were compatible or incompatible with the response to the colour (see Figure 1, from left to right): compatible direction-compatible position (CDCP), incompatible direction-compatible position (IDCP), compatible direction-incompatible position (CDIP) and incompatible direction-incompatible position (IDIP). The same numbers of trials were run for all four conditions (80 per condition). The difficulty of the task was increased by including two irrelevant dimensions to maximize the possibility of finding differences between healthy participants and the amnestic MCI subgroups [54].

After a practice block of 24 trials, a total of 320 trials (80 per condition) were presented in two blocks, with an inter-block interval of 90 s. The response button assigned to each colour of the stimulus was counterbalanced among the participants, and they were instructed to respond as quickly and accurately as possible. Half of the participants were asked to press the left button with the left hand when a red arrow appeared and the right button with the right hand when a blue arrow appeared, whereas the other half were instructed to respond in the opposite way.

### EEG Recordings

In the EEG recordings, a total of 47 active electrodes were used, in accordance with the 10–10 International System: at AFz, AF7, AF8, Fz, F3, F4, F5, F6, F7, F8, FCz, FC1, FC2, FC3, FC4, FT7, FT8, FT9, FT10, Cz, C1, C2, C3, C4, C5, C6, T7, T8, CPz, CP3, CP4, TP7, TP8, TP9, TP10, Pz, P3, P4, P7, P8, P9, P10, PO7, PO8, Oz, O1 and O2. The EEG signal was passed through a 0.01–100 Hz analog bandpass filter, and was sampled at 500 Hz. The reference electrode was placed on the tip of the nose and the ground electrode was placed at Fpz. Simultaneously to EEG recordings, ocular movement (EOG) recordings were obtained with two electrodes located supra- and infraorbitally to the right eye (VEOG) and another two electrodes at the external canthus of each eye (HEOG). All impedances were maintained below 10 kΩs. After signal storage, a two-step procedure was used to remove epochs with horizontal ocular artifacts in stimulus-locked waveforms, as carried out in previous studies [55], [56]. First, trials with large horizontal eye movements (larger than ±30 µV) were removed. Second, averaged HEOG waveforms showing residual eye movements (HEOG activity exceeding ±3 µV) were eliminated. In addition, blinks were corrected off-line by use of the algorithm of Gratton et al. (1983) [57]. The signal was passed through a 0.01–30 Hz digital band-pass filter. Epochs with signals exceeding ±100 µV were automatically rejected, and all remaining epochs were inspected individually to identify those still displaying artifacts; the epochs showing artifacts were also excluded from subsequent averaging. Epochs were then corrected to the mean voltage of the baseline (−200 to 0 in stimulus-locked ERPs, −800 to −600 in response-locked ERPs).

### Data Analyses

Trials with incorrect responses or RTs outside the 100–1200 ms range were excluded from the analysis. The RT, the magnitude of interference (defined as the difference in the RT between one condition with incompatibility of direction and/or position and the RT in the condition of double stimulus-response compatibility, i.e. the CDCP condition) and the percentage of incorrect responses were analysed.

Epochs were established between −200 and 800 ms, for waveforms associated with presentation of the stimulus (N2pc), and between −800 and 300 ms, for waveforms associated with the response (LRP-r). The mean number of averaged epochs for each experimental condition was 65 for the CG, 64 for the sdaMCI group and 61 for the mdaMCI group in stimulus-locked ERPs and 69 for the CG and 67 for the sdaMCI group and 66 for the mdaMCI group in response-locked ERPs.

To obtain the LRP-r, the difference in contralateral-ipsilateral activation for the primary motor cortex in each hemisphere was calculated. The differences were then averaged [58]. The method can be summarised by the following formula: [(C4– C3)_left hand movements_+(C3– C4)_right hand movements_]/2. Deflections with negative polarity indicate correct preparation of the response. N2pc was obtained according to the hemifield of stimulus presentation [17], as follows: [(PO8– PO7)_left hemifield_+(PO7– PO8)_right hemifield_)]/2.

The N2pc peak latency was identified as the largest negative peak between 200–375 ms after stimulus presentation. The N2pc amplitude was calculated as the averaged amplitude between 250–350 ms (based on the inspection of the grand averages and the statistics values of peak latency).

The onset latency of correct preparation of the LRP-r was analysed. The onset was determined by the method of Schwarzenau et al. (1998) [59], which assumes that the onset of correct preparation corresponds to the intersection point of two straight lines, one fitted to the baseline and another to the rising slope of the LRP. The LRP-r amplitude was obtained as the mean amplitude between −125 and −25 ms regarding the response.

The stimulus-locked lateralized readiness potential (LRP-s) was not analysed because the overlap between LRP and central contralateral negativity (N2cc) does not allow reliable measurement of the LRP-s onset [60], [61]. Nevertheless, LRP-r onset was measured because N2cc is observed at stimulus-locked averages and therefore it is jittered at response-locked averages [62].

### Statistical Analyses

With the aim of examining whether there were any differences in the RTs or the percentage of errors (PE) according to the Experimental conditions and Diagnosis, mixed measures ANOVAs were applied with two within-subject factors: Position (two levels: Compatible and Incompatible) and Direction (two levels: Compatible and Incompatible), and one inter-subject factor: Diagnosis (three levels: CG, sdaMCI, mdaMCI). A mixed measures ANOVA was conducted for the magnitude of the interference in the three conditions in which a stimulus-response incompatibility was present, with one within-subject factor: Condition (three levels: IDCP, CDIP, IDIP), and one inter-subject factor: Diagnosis (three levels: CG, sdaMCI, mdaMCI).

Mixed measures ANOVAs were applied to N2pc latency and amplitude, with two within-subject factors: Position (two levels: Compatible and Incompatible) and Direction (two levels: Compatible and Incompatible), and one inter-subject factor: Diagnosis (three levels: CG, sdaMCI, mdaMCI).

With the aim of examining possible differences in the onset latency of the preparation of the correct response in the LRP-r, as well as LRP-r mean amplitudes, corresponding mixed measures ANOVAs were carried out for each LRP parameter, with two within-subject factors: Position (two levels: Compatible and Incompatible) and Direction (two levels: Compatible and Incompatible), and one inter-subject factor: Diagnosis (three levels: CG, sdaMCI, mdaMCI).

Receiver Operating Characteristics curves (ROC, including sensitivity and specificity indexes) were calculated for those ERP parameters that showed Diagnosis to have a significant effect (i.e. N2pc and LRP amplitudes).

A Greenhouse-Geisser ε correction for the degrees of freedom was performed in all cases that the condition of sphericity was not met. As 4 mixed ANOVAs were performed for ERP data, Holm’s procedure [63] was used to constrain the Type I error while increasing the power of the test [64]. Thus, all the significant effects revealed by ANOVA were tested using Holm’s corrected α value. Measures of size effect (eta square -η^2^_ρ_-) are also provided for significant results. When the ANOVAs revealed significant effects due to the factors and their interactions, posterior comparisons of the mean values were carried out by paired multiple comparisons (adjusted to Bonferroni). Data files can be provided on request.

## Results

### Behavioral Measures

For the RT (see Table 1), the mixed measures ANOVA (Position × Direction × Diagnosis) revealed a significant effect of Position (F (1, 52) = 184.2, p<0.001, η^2^_ρ_ = 0.780), as the RT was slower when the Position was Incompatible than when it was Compatible with the required response (p<0.001). The Diagnosis factor did not reveal a significant effect in RT (F (1, 52) = 1.6, p = 0.204, p = 0.11, η^2^_ρ = _0.059).

For the percentage of errors (PE) (see Table 1), the mixed measures ANOVA (Position × Direction × Diagnosis) revealed that Diagnosis had a significant effect (F (2, 52) = 4.3, p = 0.019, η^2^_ρ = _0.141), as the PE was higher in mdaMCI than in sdaMCI (p = 0.044) and CG (p = 0.027). Position also had a significant effect (F (1, 52) = 65.7, p<0.001, η^2^_ρ_ = 0.562), as the PE was higher in trials with Incompatible Position than in trials with Compatible Position (p<0.001).

For the magnitude of the interference, the mixed measures ANOVA (Interference × Diagnosis) revealed that the type of Interference had a significant effect (F (2, 104) = 85.7, p<0.001, η^2^_ρ_ = 0.622), as the interference was greater in CDIP than in IDCP (p<0.001), and it was greater in IDIP than in IDCP (p<0.001). Diagnosis did not exert significant effects.

### ERPs

**Figure 2:**
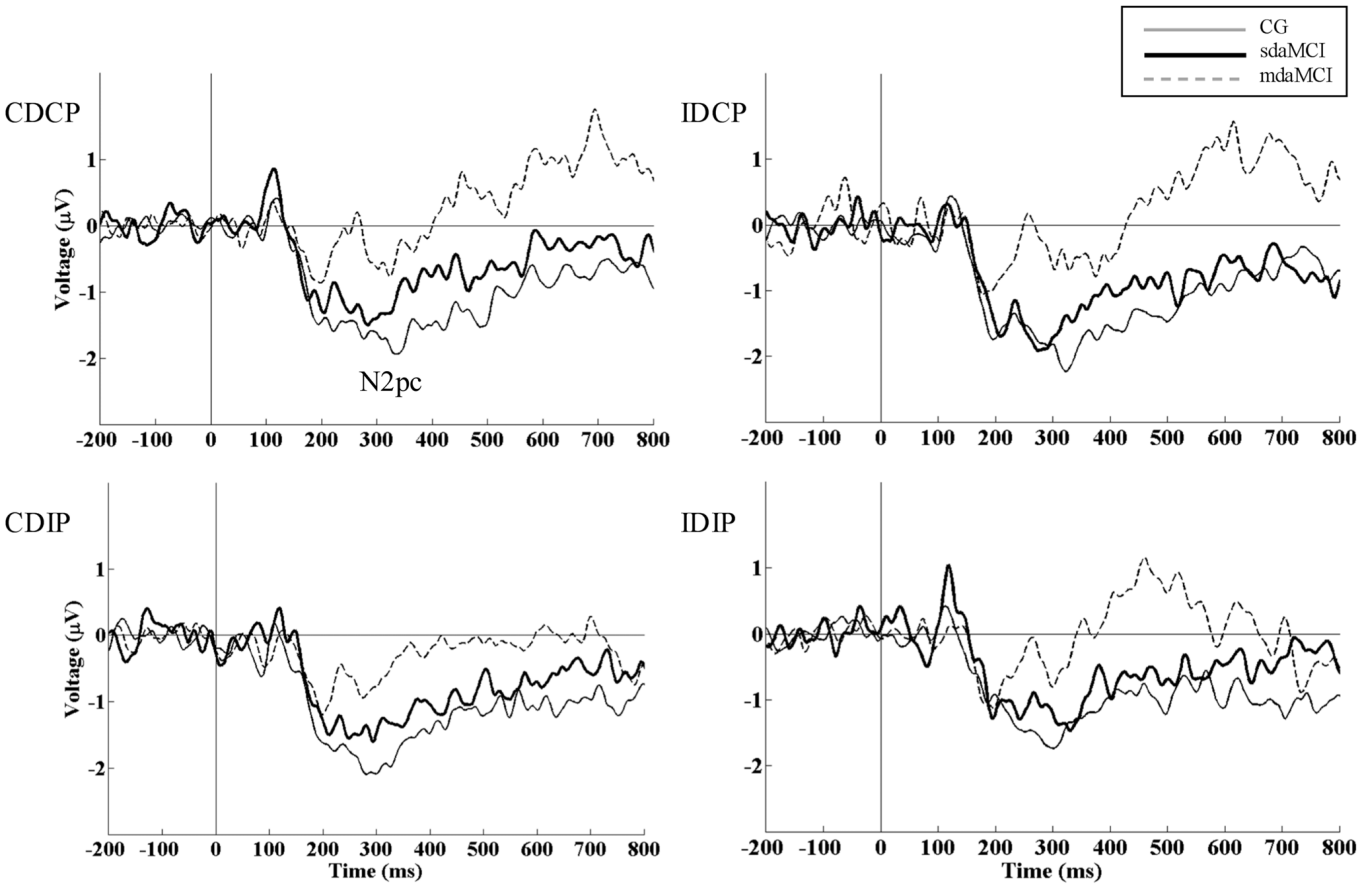
Group-related effects in N2 posterior contralateral (N2pc). The N2pc at the PO7/PO8 electrode pair is represented for the CG (thin solid line), single-domain amnestic MCI (sdaMCI) (thick solid line) and multiple-domain amnestic MCI (mdaMCI) (dashed line) groups in the four conditions of the task (CDCP, IDCP, CDIP and IDIP). N2pc amplitude was smaller in mdaMCI patients than in CG, suggesting reduced visuospatial processing in mdaMCI participants. No differences among groups in N2pc latency were observed.

**Figure 3:**
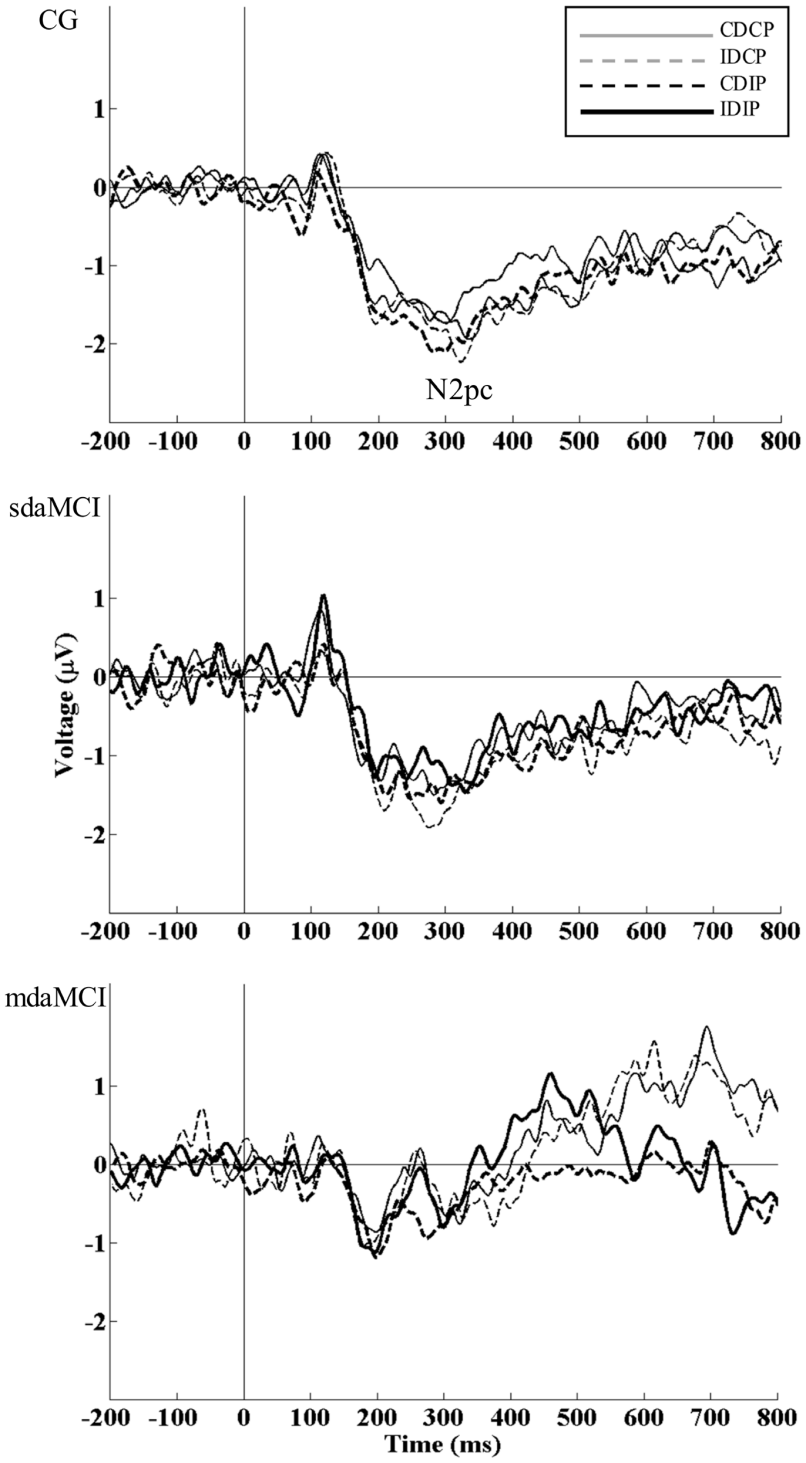
Condition-related effects in negativity posterior contralateral (N2pc). The N2pc at the PO7/PO8 electrode pair is represented for each of the groups (CG (top), sdaMCI group (middle), and mdaMCI group (bottom) for each experimental condition: CDCP (solid gray line), IDCP (dashed gray line), CDIP (dashed black line), and IDIP (solid black line). As in previous studies with elderly participants, no effects related to the experimental conditions were observed on the N2pc parameters.

For the N2pc latency, the mixed measures ANOVA (Position × Direction × Diagnosis) did not reveal any significant effects. For the N2pc amplitude, the mixed measures ANOVA (Position × Direction × Diagnosis) revealed that Diagnosis had a significant effect (F (1, 52) = 4.8, p = 0.013, η^2^_ρ_ = 0.155), as the N2pc amplitude was smaller in the mdaMCI than in the CG participants (p = 0.011) (see Table 2 and Figure 2). Also, the mixed measures ANOVAs did not reveal effects from the experimental manipulations (see Figure 3).

**Table 2:** Mean and standard deviation, for each Condition (Compatible Direction-Compatible Position (CDCP), Incompatible Direction-Compatible Position (IDCP), Compatible Direction-Incompatible Position (CDIP) and Incompatible Direction-Incompatible Position (IDIP)) and group (Control Group (CG), single-domain amnestic Mild Cognitive Impairment (sdaMCI), and multiple-domain amnestic Mild Cognitive Impairment (mdaMCI) of Reaction Time (in milliseconds); Percentage of Errors (PE); peak latency and averaged amplitude (measured as averaged amplitude between 250–350 ms) of N2pc at PO7/PO8 electrodes pair; response-locked lateralized readiness potential (LRP-r) onset and LRP-r amplitude (averaged amplitude between −125– −25 ms) at C3/C4 electrodes pair.

	RT	PE	N2pclat	N2pcamp	LRPonset	LRPamp
**CG CDCP**	544 (86)	2.6 (3.7)	300 (43)	−2.0 (1.5)	−311 (75)	−5.6 (1.9)
**CG IDCP**	547 (79)	2.6 (3.2)	300 (31)	−2.2 (1.8)	−294 (59)	−5.7 (1.7)
**CG CDIP**	595 (85)	7.4 (5.2)	291 (37)	−2.2 (2.0)	−239 (44)	−5.3 (1.9)
**CG IDIP**	590 (86)	5.3 (3.7)	287 (43)	−1.7 (1.6)	−244 (40)	−5.3 (2.0)
**sdaMCI CDCP**	580 (109)	2.4 (3.0)	306 (43)	−1.3 (1.2)	−342 (84)	−3.8 (1.8)
**sdaMCI IDCP**	593 (126)	2.5 (3.5)	292 (23)	−1.7 (1.1)	−311 (74)	−4.0 (1.6)
**sdaMCI CDIP**	631 (123)	6.6 (5.8)	296 (43)	−1.4 (1.3)	−279 (6.6)	−3.4 (1.3)
**sdaMCI IDIP**	634 (140)	6.5 (5.3)	314 (37)	−1.2 (1.0)	−271 (70)	−3.6 (1.6)
**mdaMCI CDCP**	595 (97)	5.1 (5.4)	304 (45)	−0.5 (1.1)	−310 (80)	−4.0 (1.8)
**mdaMCI IDCP**	593 (91)	5.7 (4.9)	320 (38)	−0.7 (1.1)	−316 (76)	−4.1 (1.9)
**mdaMCI CDIP**	664 (121)	11.2 (7.1)	303 (34)	−0.9 (1.2)	−286 (93)	−3.6 (2.0)
**mdaMCI IDIP**	651 (94)	11.3 (8.2)	310 (36)	−0.6 (1.3)	−280 (71)	−3.6 (2.0)

**Figure 4:**
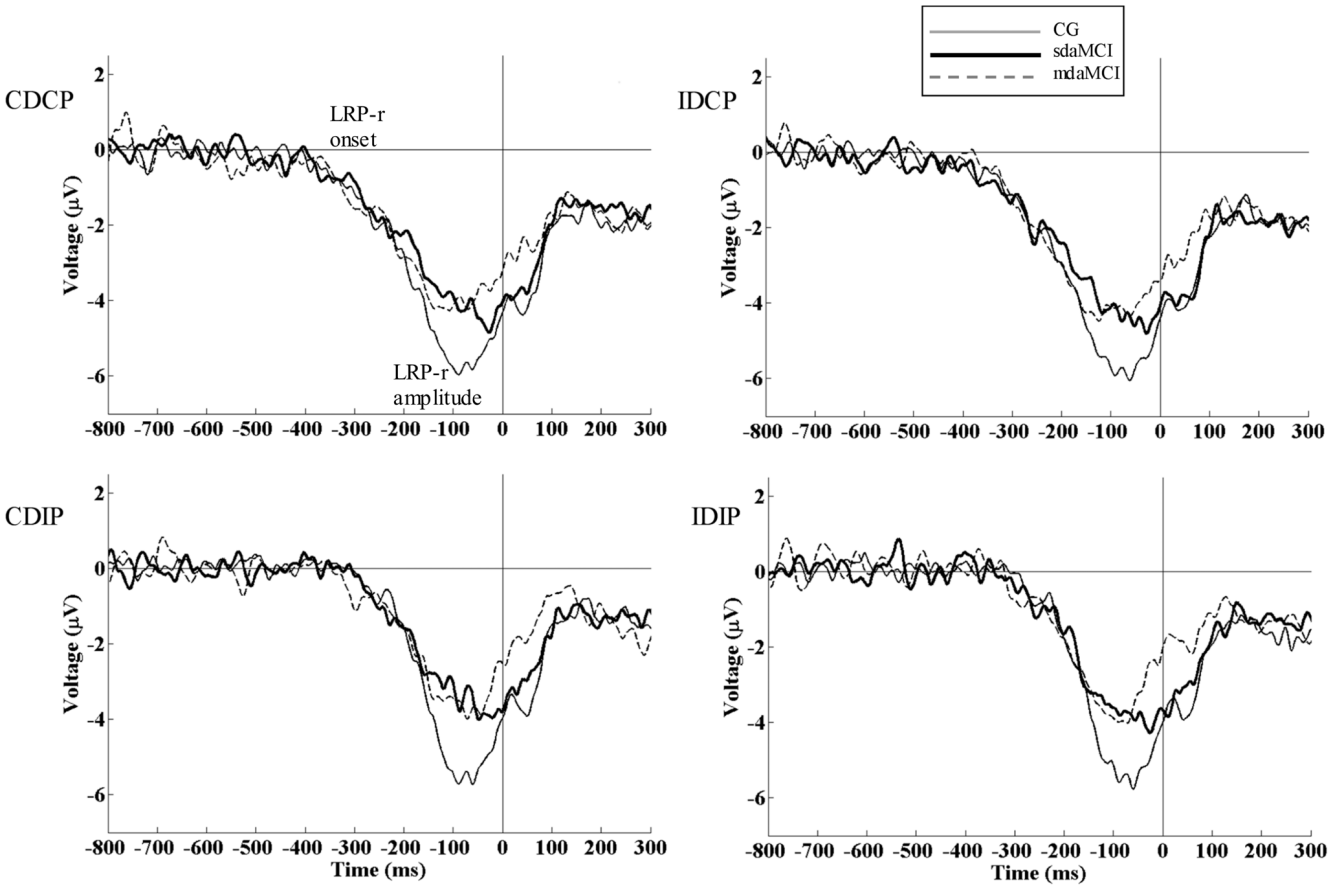
The response-locked lateralized readiness potential (LRP-r). The LRP-r is represented for the CG (thin solid line), single-domain amnestic MCI (sdaMCI) (thick solid line) and multiple-domain amnestic MCI (mdaMCI) (dashed line) groups in the four conditions of the task (CDCP, IDCP, CDIP and IDIP). The LRP-r onset latency (the point where starts the negative trend in the waveform) and LRP-r mean amplitude (−125 – −25 ms) were calculated. The LRP-r amplitude was larger in healthy participants than in sdaMCI and mdaMCI groups, suggesting declined mechanisms for implementing the response in sdaMCI and mdaMCI patients.

**Figure 5:**
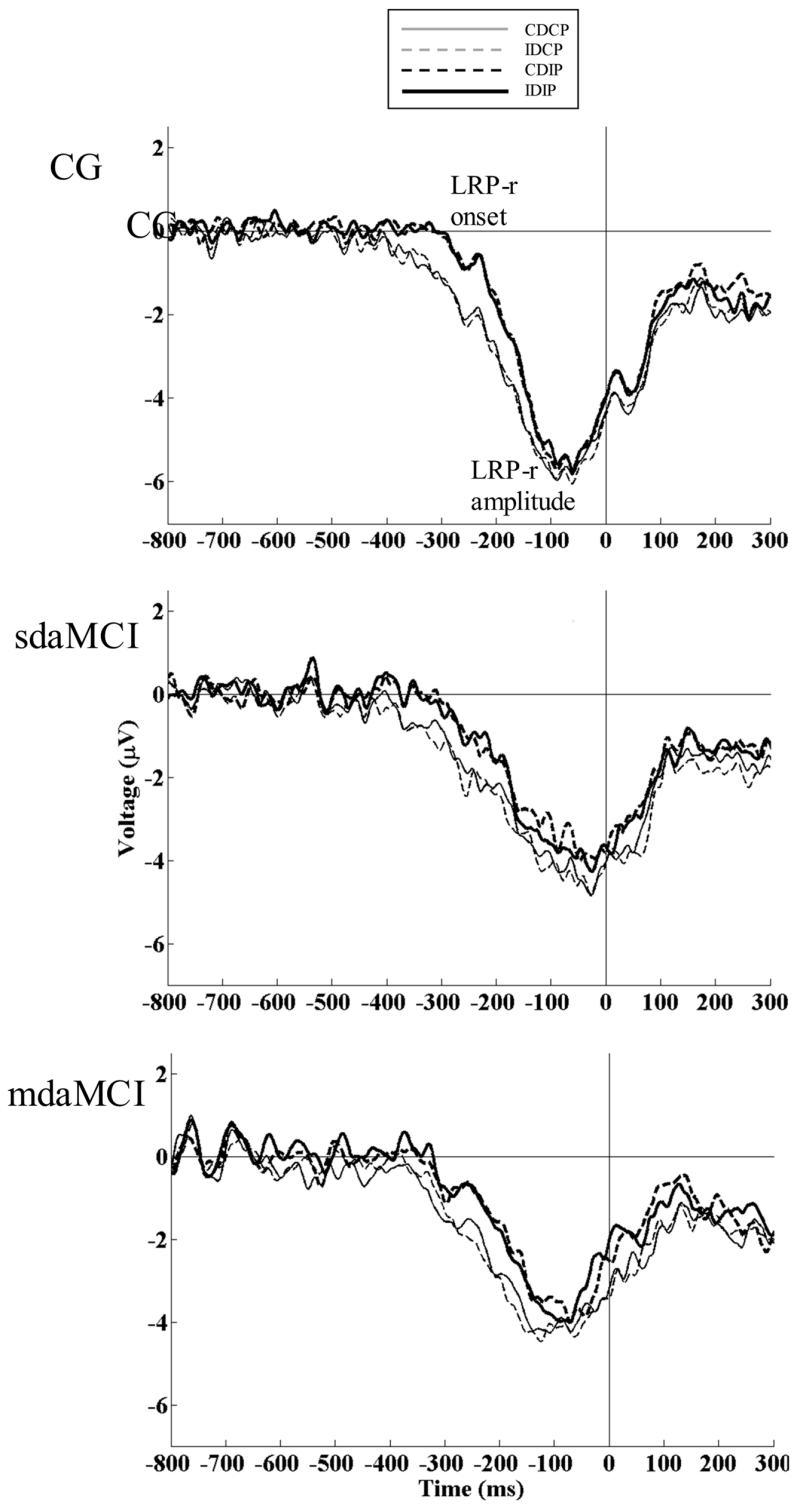
Condition-related effects in response-locked lateralized readiness potential (LRP-r). The LRP-r at the C3/C4 electrode pair is represented for each of the groups (top: CG, middle: sdaMCI group, bottom: mdaMCI group) in each experimental condition (CDCP (solid gray line), IDCP (dashed gray line), CDIP (dashed black line), and IDIP (solid black line). When stimulus position is incompatible with the required response (CDIP, IDIP), a delay in the preparation of the correct response is observed in the three groups of participants. This result is consistent with previous studies demonstrating an interference locus for incompatibility from the stimulus position at the response-execution stage.

The mixed measures ANOVA (Position × Direction × Diagnosis) for the LRP-r mean amplitude revealed that Diagnosis had a significant effect (F (1, 52) = 7.1, p = 0.002, η^2^_ρ_ = 0.214), as the LRP-r amplitude was larger in CG than in the sdaMCI group (p = 0.005) and mdaMCI group (p = 0.019) (see Table 2 and Figure 4). Position also had a significant effect (F (1, 50) = 9.6, p = 0.003, = η^2^_ρ_ 0.155), as the amplitude was larger when the position was Compatible than when it was Incompatible with the response (p = 0.003) (see Figure 5). Regarding the LRP-r onset latency, the mixed measures ANOVA (Position × Direction × Diagnosis) showed that Position had a significant effect (F (1, 52) = 38.1, p<0.001, η^2^_ρ_ = 0.423), as the LRP-r onset was earlier when the Position was Compatible than when it was Incompatible with the required response (p<0.001) (see Figure 5).

**Figure 6:**
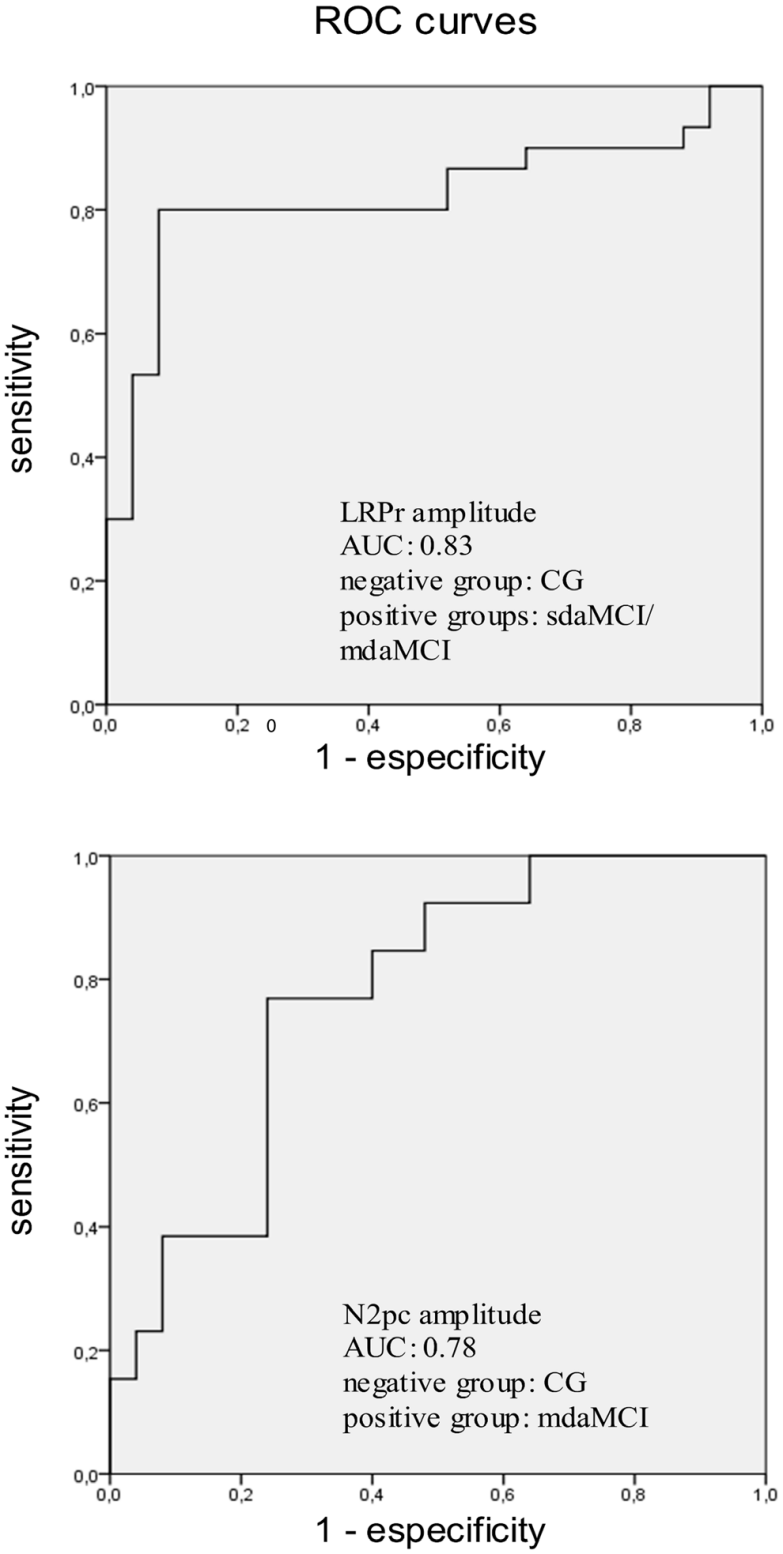
Single domain amnestic MCI (sdaMCI) and multi domain amnestic MCI (mdaMCI) correlates and biomarkers. Receiver operating characteristics curves (ROC) are represented for LRP amplitude (top) and N2pc amplitude (bottom). Indexes of sensitivity and specificity, and area under curve (AUC) are reported for LRP and N2pc amplitudes respectively. For the LRP amplitude, the selected cut-off was −3.75 µV (sensitivity: 0.80, specificity: 0.92). For the N2pc amplitude, the selected cut-off was −1.11 µV (sensitivity: 0.77, specificity: 0.76).

ROC analysis (negative group: GC; positive group: mdaMCI) for N2pc amplitude (see Figure 6, bottom panel) revealed an area under curve (AUC) of 0.78. Using the value of −1.11 µV as a cut-off, the indexes of sensitivity and specificity were 0.77 and 0.76 respectively. ROC analysis (negative group: CG; positive groups: sdaMCI and mdaMCI groups) for LRP-r amplitude (see Figure 6, top panel) yielded an AUC of 0.82. Using the value of −3.75 µV as a cut-off, the sensitivity and specificity indexes were 0.80 and 0.92 respectively.

## Discussion

The aim of the present study was to search for ERP biomarkers of single-domain amnestic MCI (sdaMCI) and multiple-domain amnestic MCI (mdaMCI) groups by studying healthy elderly and sdaMCI and mdaMCI groups while they performed a Simon task. The main results were as follows: a) the RT and the interference effect did not differ between CG, sdaMCI, and mdaMCI groups. However, the percentage of errors (PE) was higher in mdaMCI than in sdaMCI and CG; b) the N2pc amplitude was smaller in mdaMCI than in CG, thus constituting a biomarker of mdaMCI, with an area under curve (AUC) of 0.78; c) The LRP-r amplitude was smaller in the two amnestic MCI subgroups (sdaMCI and mdaMCI) than in CG, constituting a biomarker of the two amnestic MCI subgroups, with an AUC of 0.83.

The Reaction time (RT) and the interference effect did not differ between healthy and MCI groups. However, differences between CG and mdaMCI group were found in the PE. This result may be associated with an incipient decline in monitoring the selection of the correct response in the mdaMCI group, which may be related to impaired executive functions in that group. Results of previous studies concerning inhibitory control in MCI patients show highly variable results (i.e., some studies report preserved inhibitory control [65]–[67], whereas other studies show a decline in inhibitory control in MCI patients [35], [36]). These discrepancies are probably related to the heterogeneous sample characteristics (e.g., differences in standard deviations for considering existence of impairment in cognitive tests, different MCI subtypes included on each study) and the experimental tasks used.

The position of the arrow caused a Simon effect (longer RT and higher PE when it was incompatible with the response side). This is consistent with previous findings for samples of young [60], [68] and elderly [38] participants. However, interference from the direction (in IDCP condition) was not significant. This result was inconsistent with previous results in a sample of young adults performing an identical task [56]. Nonetheless, in the latter study interference from the position was greater than interference from the direction, as the stimulus position attracts attentional resources more automatically and rapidly than the direction [53], [69], [70], which would partially mask the effect of the direction. In the present study, it is possible that a greater age-related decline for effortful than for automatic processes [71] increased the above masking and nullified the direction effect, as would be consistent with results obtained in samples of healthy middle-aged and elderly participants performing an identical task [72]. On the other hand, effect of the position-direction interaction (in the IDIP condition) was not significant, as also found in previous studies [56], [73].

Electrophysiological measures showed that the motor response execution stage was not longer in sdaMCI and mdaMCI patients than in healthy participants (i.e. differences in LRP-r onset were not present), which is consistent with the absence of any differences in reaction times between both groups. On the other hand, the incompatibility of the position delayed the LRP-r onset, demonstrating interference from this irrelevant dimension at the response execution stage, as previously suggested on the basis of behavioral [74], [75] and ERP [69] data.

The amplitude of the LRP-r was smaller in sdaMCI and mdaMCI patients than in healthy participants. As far as we know, this is the first study focusing on LRP amplitudes in samples of MCI patients, and consequently the first report of smaller LRP amplitudes in sdaMCI and mdaMCI than in healthy participants. Importantly, the LRP-r amplitude may be of clinical interest from a diagnostic point of view, since it yielded good indexes of sensitivity and specificity, 0.80 and 0.92 respectively, for a cut-off of 3.75 µV (see top panel of the Figure 6). It is important to note that the LRP is obtained by a non-invasive procedure through a relatively inexpensive and widely used technique, i.e., the ERP.

In previous studies, larger LRP amplitudes in healthy elderly participants than in young participants have been attributed to reduced inhibitory control [30]. However, larger LRP amplitudes were found when the stimulus position was compatible with the response, and shorter RT and lower PE were observed. In other words, larger LRP amplitudes were associated with behavioural indexes of better inhibitory control. Also, consistent with this observation, the smaller LRP amplitudes in sdaMCI and mdaMCI patients may be related to incipient impairment of electrophysiological correlates of implementation of motor resources for executing the response, which would still not be manifested in the behavioural performance (although a higher PE was found in mdaMCI than in sdaMCI and CG). This interpretation is consistent with recent reports of deficits in motor regions in amnestic MCI patients, observed in transcranial magnetic stimulation (TMS) studies [33], [76], [77]. Thus, the results of the present study are consistent with the view of amnestic (the two amnestic subtypes, single- and multiple-domain) MCI patients showing deficits in the motor cortex, as revealed by LRP-r amplitude, which may also constitute an early electrophysiological marker of sdaMCI and mdaMCI states.

The timing of visuospatial processing of the target stimulus (represented by the ERP correlate N2pc latency), did not reveal any differences between participants according to the diagnosis. No previous studies have focused on N2pc latency in samples of MCI patients. Therefore, on the basis of the evidence of the present results, it can be concluded that the speed of attentional shifts to target stimuli is not affected in sdaMCI and mdaMCI patients because N2pc peak latency did not differ between groups.

The N2pc amplitude was smaller in mdaMCI patients than in healthy participants, a result that suggests a reduced allocation of attentional resources to the target stimulus in the mdaMCI patients. Therefore, mdaMCI patients may have some impairments in the brain areas that generate the N2pc component, basically temporal and parieto-occipital regions (for details on the N2pc sources see Hopf et al., 2000 [78] and Lorenzo-López et al., 2011 [25]). This result is consistent with behavioural evidence for declined visuospatial abilities in samples of MCI patients [11]. However, some authors have suggested that visuospatial deficits may take place earlier than the typical memory impairments observed in early stages of the AD [39], [40]. The results of the present study using a Simon task did not provide any evidence supporting the above affirmation because there were no differences in correlates of visuospatial processes (i.e., in N2pc latency and amplitude) between healthy elderly and sdaMCI group. This is also consistent with the absence of differences between CG and sdaMCI groups in behavioural data.

ROC analyses showed that CG and mdaMCI group were moderately well distinguished on the basis of N2pc amplitude. This analysis yielded an AUC of 0.78 (0.77 of sensitivity and 0.76 of specificity for a cut-off of –1.11 µV; see the bottom panel of Figure 6). Therefore, as a result of the decline in memory accompanied by decline in other cognitive functions, it is more likely that correlates of visuospatial processes (N2pc amplitude) in a Simon task will differ from those in healthy participants. This may be associated with greater progression of pathophysiological processes in mdaMCI than in sdaMCI participants, as suggested in previous studies [11], [79].

Sets of neuropsychological tests that commonly include (among other aspects) evaluation of memory functions do not usually show higher sensitivity and specificity values than those observed in the present study for N2pc (0.77 and 0.76 sensitivity and specificity, respectively) and LRP amplitude (0.80 and 0.92 of sensitivity and specificity, respectively) (for reviews see Lonie et al., 2009 [80]; Mora-Simón et al., 2012 [81]). Therefore, the present investigation may represent a first step in establishing N2pc –in sdaMCI- and/or LRP-r –in sdaMCI and mdaMCI- amplitudes as early biomarkers of amnestic MCI. As far as we know, this is the first time that sensitivity and specificity of visuospatial and motor ERP biomarkers are obtained for amnestic MCI patients. It is noticeable that these markers provide comparable measures to those obtained for episodic memory [82], [83]. Therefore, the present results encourage further research to replicate and confirm these preliminary findings. In addition, in order to test if N2pc and LRP amplitudes constitute good predictors of conversion to Alzheimer disease, future studies should include a sample of AD patients, and/or groups of sdaMCI and mdaMCI converting to AD patients compared to those that remain as amnestic MCI patients.

Some studies have shown that N2pc may reflect activity related to processing of the target as well as suppression of the non-target [18]. Thus, the previously mentioned deficits in mdaMCI participants may be related to target processing and also to suppression of the non-target stimulus. However, when a single contralateral non-target appears in the display (as in the Simon task of the present study), the N2pc waveform basically reflects activity related to target processing [19]. This is further suggested by considering the distance between target and non-target in the task used in the present study (7.5°). Since receptive fields in the extrastriate cortex are comprised between 3°–8° of visual angle [84], competition between target and non-target is unlikely to occur. Thus, the decreased N2pc amplitude observed in mdaMCI patients was probably due only to impairment in the allocation of attentional resources to the target stimulus.

Finally, in a previous study in which young adults performed an identical task [56], N2pc was modulated by a conflict of spatial information conveyed by both irrelevant dimensions (i.e., N2pc was smaller when the direction of the arrow pointed to the opposite side regarding the hemifield where it was located). In the present study, the absence of N2pc amplitude modulations can be easily explained as there were no directional effects. Moreover, the N2pc was not modulated by interference from the stimulus position, which is consistent with other reports [26], [61].

## Conclusions

In summary, the present study investigated visuospatial and motor correlates of two amnestic MCI subtypes during the performance of a Simon task by 25 healthy participants: 17 single-domain MCI (sdaMCI) and 13 multiple-domain MCI (mdaMCI) patients. No behavioural differences between healthy and sdaMCI participants were found, and only a higher percentage of errors was observed for mdaMCI relative to healthy participants. However, electrophysiological correlates of cognitive processes showed an incipient decline in the two groups of amnestic MCI patients, indicating that changes in brain may start earlier than the changes in behavioural performance. Regarding visuospatial processes, the speed of attentional shifts to the target stimulus (N2pc latency) was similar in the two subtypes of amnestic MCI patients and healthy participants. However, the N2pc amplitude, an index of the amount of attentional resources devoted to the target stimulus, was smaller in the multiple-domain amnestic MCI group than in the control group, which suggests a decline in the neural sources of the N2pc component (i.e., in temporal and parieto-occipital regions) in the mdaMCI group. ROC analyses of N2pc amplitude including CG and mdaMCI groups revealed sensitivity and specificity indexes of of 0.77 and 0.76, respectively. Moreover, the time of response execution was not extended in sdaMCI and mdaMCI participants, which is consistent with the absence of significant differences in RTs. However, the LRP-r amplitude was smaller in sdaMCI and mdaMCI patients than in healthy participants, suggesting impairment of frontal motor areas. Furthermore, the ROC curves provided preliminary data supporting utility of LRP-r amplitude as a good biomarker of sdaMCI and mdaMCI (indexes of sensitivity and specificity were 0.80 and 0.92, respectively).
